# Phenotypic characterization of *Adig* null mice suggests roles for adipogenin in the regulation of fat mass accrual and leptin secretion

**DOI:** 10.1016/j.celrep.2021.108810

**Published:** 2021-03-09

**Authors:** Anna Alvarez-Guaita, Satish Patel, Koini Lim, Afreen Haider, Liang Dong, Olivia J. Conway, Marcella K.L. Ma, Davide Chiarugi, Vladimir Saudek, Stephen O’Rahilly, David B. Savage

**Affiliations:** 1 Metabolic Research Laboratories, Wellcome Trust-Medical Research Council Institute of Metabolic Science, University of Cambridge, Cambridge, Cambridgeshire CB2 0QQ, UK; 2 MRC Metabolic Diseases Unit, University of Cambridge Metabolic Research Laboratories, Wellcome Trust-MRC Institute of Metabolic Science, Genomics and Transcriptomics Core, Addenbrooke’s Hospital, Cambridge CB2 0QQ, UK

**Keywords:** adipogenin, adipose tissue, adipogenesis, leptin, knockout mouse

## Abstract

Adipogenin (*Adig*) is an adipocyte-enriched transmembrane protein. Its expression is induced during adipogenesis in rodent cells, and a recent genome-wide association study associated body mass index (BMI)-adjusted leptin levels with the *ADIG* locus. In order to begin to understand the biological function of *Adig*, we studied adipogenesis in *Adig*-deficient cultured adipocytes and phenotyped *Adig* null (*Adig*^*−/−*^) mice. Data from *Adig*-deficient cells suggest that *Adig* is required for adipogenesis. *In vivo*, *Adig*^*−/−*^ mice are leaner than wild-type mice when fed a high-fat diet and when crossed with *Ob/Ob* hyperphagic mice. In addition to the impact on fat mass accrual, *Adig* deficiency also reduces fat-mass-adjusted plasma leptin levels and impairs leptin secretion from adipose explants, suggesting an additional impact on the regulation of leptin secretion.

## Highlights

• The absence of Adig impairs adipogenesis *in vitro*
• High-fat diet (HFD)-induced weight gain is ameliorated in *Adig*^*−/−*^ mice
• Fat-mass-adjusted leptin levels are reduced in *Adig*^*−/−*^ mice
• Leptin secretion is reduced in *Adig*^*−/−*^ adipose explants

## Graphical abstract

**Figure:**
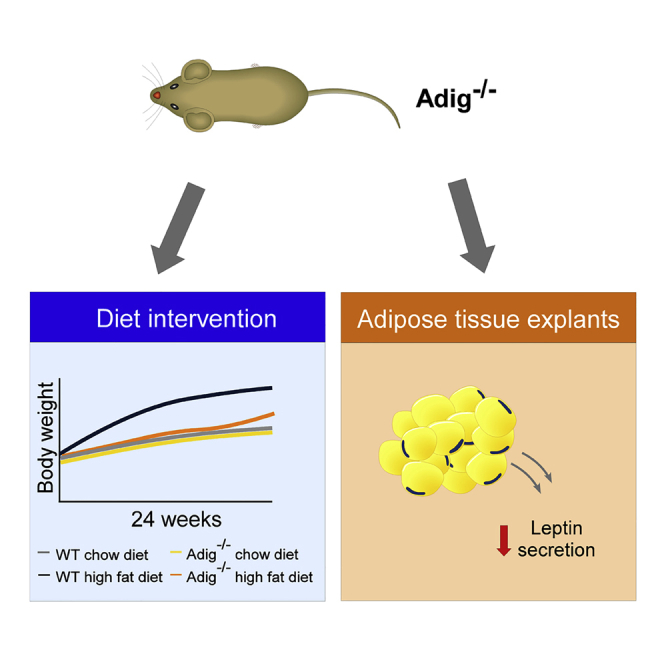

## Teaser

Alvarez-Guaita et al. show that Adipogenin (*Adig*) deficiency impairs adipogenesis in cultured cells. High-fat diet (HFD)-induced weight gain is ameliorated in *Adig*^*−/−*^ mice, and fat-mass-adjusted leptin levels are lower. Leptin secretion from *Adig*^*−/−*^ adipose explants is also reduced, suggesting that Adig influences leptin secretion.

## Introduction

Adipogenin (*Adig*), also known as SMAF1 for Small Adipocyte Factor 1, is a small (∼10 kDa) membrane protein (Hong et al., 2005; Kim et al., 2005). *Adig* mRNA was originally detected in the liver (Yu et al., 2003), but two independent groups subsequently showed that its expression is induced during adipocyte differentiation in 3T3-L1 cells and that knocking it down affects this process (Hong et al., 2005; Kim et al., 2005). In mice, *Adig* was reported to be predominantly expressed, both at the mRNA and protein level in the adipocyte fraction of adipose tissue (AT) and was also found to have a PPARγ response element in its promoter (Ren et al., 2016a). Initial subcellular localization studies suggested that it might localize to the nucleus, lipid droplets, or membranes, although these studies were all conducted using overexpressed tagged proteins (Hong et al., 2005; Kim et al., 2005; Ren et al., 2016a).

Our interest in *Adig* was triggered by the discovery that a locus near the human *ADIG* gene was shown to be associated with body mass index (BMI)-adjusted leptin levels (Kilpeläinen et al., 2016). Leptin is almost exclusively expressed in adipocytes, and its plasma concentration is strongly associated with fat mass and BMI, but despite its fundamental importance in energy balance homeostasis, exactly how its expression and secretion are regulated *in vivo* remains unclear (Flier and Maratos-Flier, 2017; Friedman, 2014; Pan and Myers, 2018). The genome-wide association study (GWAS) performed by Kilpeläinen et al. (2016) involved data from 32,161 individuals and suggested that a SNP (rs6071166) near the *SLC32A1* locus was associated with BMI-adjusted leptin concentrations. Although the SNP was not associated with mRNA expression of nearby genes in AT, liver, lymphocytes, brain, or skin, the authors measured the expression levels of murine homologous genes surrounding the variant in mouse AT. This analysis identified *Adig* as a candidate gene in the *SLC32A1* locus, as it was relatively highly expressed compared to other nearby genes. Moreover, knockdown (KD) of *Adig* in epididymal AT explants decreased leptin expression and secretion by ∼25% (Kilpeläinen et al., 2016).

Although the therapeutic use of leptin has, to date, been limited to rare leptin-deficient states including monogenic forms of obesity associated with bialleleic *LEP* mutations, generalized, and to a lesser extent, partial lipodystrophies and hypogonadotrophic hypogonadism in very lean females, there is growing interest in the potential to use leptin therapeutically in at least some more prevalent states such as “low-leptin-associated obesity” and low-leptin-associated non-alcoholic fatty liver disease (NAFLD) (Dallner et al., 2019; Friedman, 2016). So, understanding how its secretion is regulated remains very important.

Here, we sought to explore the *in vivo* function of *Adig* in AT by phenotypically characterizing *Adig* null mice, as well as revisiting the impact of *Adig* KD in cultured pre-adipocytes.

## Results

### Confirmation of *Adig* deletion in mice and impact on body weight

**Figure 1:**
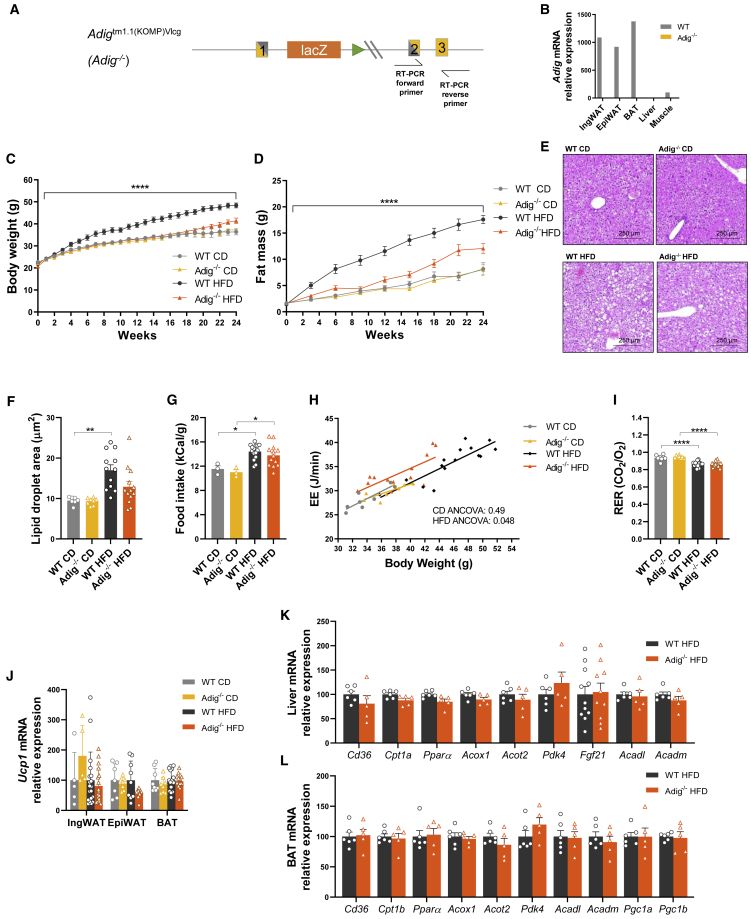
Confirmation of *Adig* deletion in mice and impact on body weight (A) Schematic illustration of the *Adig* knockout strategy, which results in a loss of parts of exons 1 and 2. RT-PCR primer sites used to quantify relative *Adig* mRNA expression are included. (B) *Adig* mRNA expression in tissues from 10-week-old male WT (wild-type) and *Adig*^−/−^ mice. Expression is presented relative to WT muscle (set at 100) (n = 1). (C and D) WT and *Adig*^−/−^ male mice (aged 5 weeks) were fed a chow diet (CD) or high-fat diet (HFD) for 24 weeks, during which time their body weights were recorded weekly (C) and their fat mass every 3–4 weeks (D) (n = 14–21). (E and F) (E) Representative hematoxylin/eosin liver images (250 µm scale bar) and (F) lipid droplet size analysis in liver samples at the end of the experiment (n = 7-13). (G) Food intake recorded over 2 weeks in WT and *Adig*^−/−^ male mice after 22 weeks on a CD (n = 3) or HFD (n = 13–15). (H) Energy expenditure (EE) expressed relative to the body weight of each individual mouse fed a CD or HFD for 21 weeks and then analyzed using 2-way ANCOVA (n = 8–15). (I) Respiratory exchange ratio (RER) (n = 8–15). (J) Relative *Ucp1* mRNA expression in IngWAT, EpiWAT, and BAT at the end of the experiment (24 weeks CD or HFD) relative to WT (set at 100) (n = 5–19). (K and L) Cd36, Cpt1a, Pparα, Acox1, Acot2, Pdk4, Fgf21, Acadl, and Acadm mRNA expression in liver (n = 5-11) (K) and Cd36, Cpt1b, Pparα, Acox1, Acot2, Pdk4, Acadl, Acadm, Pgc1a, and Pgc1b mRNA expression in BAT samples (L) taken after 24 weeks on a HFD relative to WT HFD (set at 100) (n = 5–6). Data are expressed as mean ± SEM and were analyzed by 2-way ANOVA (C and D; p value obtained from the interaction of genotype and diet), 1-way ANOVA with Bonferroni multiple-comparison post hoc testing (F, G, I, and J), ANCOVA (H), or two-tailed Student’s t tests (K and L). ^∗^p < 0.05, ^∗∗^p < 0.01, ^∗∗∗^p < 0.001, ^∗∗∗∗^p < 0.0001. IngWAT, inguinal white adipose tissue; EpiWAT, epididymal WAT; MesWAT, mesenteric WAT; RetroWAT, retroperitoneal WAT; BAT, brown AT.

*Adig* null mice were generated by *in vitro* fertilization using sperm from the *Adig*^tm1.1(KOMP)Vlcg^ line (KOMP, UC Davis), which lacks part of exons 1 and 2 of the *Adig* gene (Figure 1A). Heterozygous *Adig*^tm1.1(KOMP)Vlcg^ mice were backcrossed onto pure C57BL/6J mice in order to generate heterozygous *Adig*^tm1.1 (KOMP)Vlcg^ mice. Subsequently, *Adig*^+/−^ mice were bred, and the resultant *Adig*^+/+^ (wild type; WT) and *Adig*^−/−^ littermates were used for metabolic phenotyping.

*Adig* deletion was confirmed by RT-PCR (Figure 1A) in mouse inguinal (IngWAT) and epididymal (EpiWAT) white AT (WAT), brown AT (BAT), and other tissues such as the liver and skeletal muscle, where *Adig* mRNA expression is already extremely low in WT mice (Figure 1B). The *Adig*^−/−^ mice did not manifest any obvious morphological phenotypes and were viable and fertile. When fed a chow diet (CD), both male and female *Adig* knockouts were similar to WT littermates in terms of weight gain and fat and lean mass up to age ∼30 weeks (Figures 1C and 1D, S1A, and S1C). However, when fed a high-fat diet (HFD; 45% fat), *Adig*^−/−^ littermates diverged from the WT mice from 4 weeks onward, maintaining a similar body weight and fat mass to CD-fed mice (Figures 1C and 1D). This result persisted until about 20 weeks of HFD exposure when the *Adig*^−/−^ group gained more weight than CD-fed mice, although their weight remained significantly lower than that of WT HFD-fed mice (Figure 1C). These changes in body weight were largely dependent on differences in fat mass, whereas lean mass remained similar in all groups (Figures 1D and S1A). The increased fat mass in *Adig*^−/−^ mice fed a HFD was apparent in both subcutaneous (inguinal) and visceral (epididymal, mesenteric, and retroperitoneal) fat depots, whereas BAT weights were similar in both genotypes (Figures S1B and S1D). Liver weights were similar in both groups when fed a CD but increased to a greater extent in WT mice fed a HFD, which is in line with the overall changes in body weight and fat mass (Figures S1B and S1D). Liver steatosis was apparent in HFD-fed mice of both genotypes (Figure 1E), and average lipid droplet area corresponded with the differences in liver weight (Figure 1F).

In order to understand the mechanisms underpinning the differences in body weight, food intake and energy expenditure (EE) were assessed in male mice after 21 weeks on a CD and HFD. Both WT and *Adig*^−/−^ mice showed similar energy intake on a CD, and this increased to a similar extent on a HFD (Figure 1G). EE, assessed using indirect calorimetry over 48 h, was similar in mice fed a CD, whereas the analysis of covariance (ANCOVA) results suggested that it was elevated in the *Adig*^−/−^ mice on a HFD (Figure 1H; CD ANCOVA: p = 0.49, HFD ANCOVA: p = 0.048). Fat oxidation increased in both groups when fed a HFD but was otherwise similar between WT and *Adig*^−/−^ mice, as reflected in the respiratory exchange ratio (RER) (Figure 1I).

Given the differences in EE, we also assessed BAT mass and *Ucp1* expression in the *Adig*^−/−^ mice. As shown in Figure S1B, BAT mass was similar in both genotype groups, and *Ucp1* mRNA expression was similar in BAT as well as in both IngWAT and EpiWAT regardless of the diet (Figure 1J), suggesting that the observed increase in EE may not be mediated by BAT or secondary to browning of WAT depots, although the data do not formally exclude these possibilities. Evaluation of the expression of genes typically involved in BAT and hepatic fat oxidation was also similar in WT and *Adig*^−/−^ mice (Figures 1K and 1L).

### Biochemical assessment of glucose and insulin tolerance in *Adig*^*−/−*^ mice

**Figure 2:**
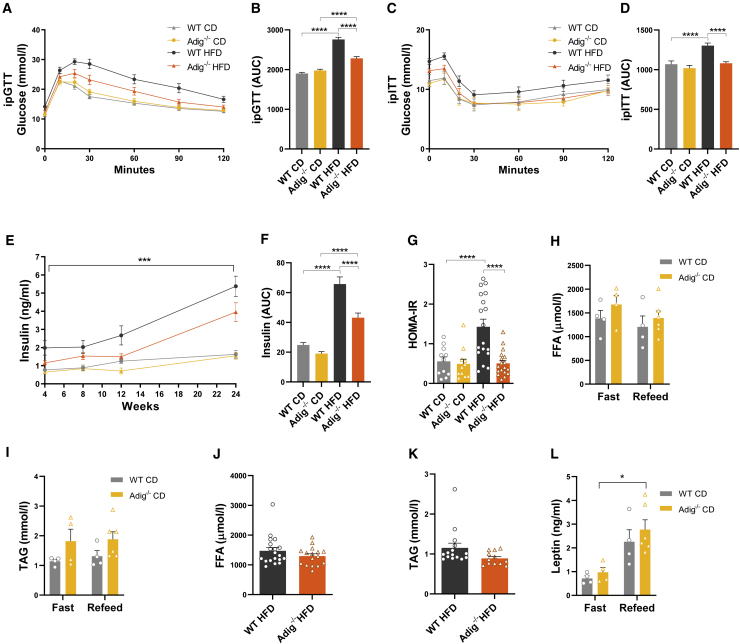
Biochemical assessment of glucose and insulin tolerance in *Adig*^*−/−*^ mice (A and C) Blood glucose concentrations during intraperitoneal glucose and insulin tolerance tests (ipGTT and ipITT, respectively) undertaken in WT and *Adig*^−/−^ male mice after 16 and 19 weeks fed CD and HFD, respectively. (B and D) Area under the curve (AUC) data for the ipGTT and ipITT (n = 9–18). (E and F) Random fed plasma insulin concentrations in samples taken at the indicated time points during 24 weeks on a CD or HFD in WT and *Adig*^−/−^ male mice and the AUC (n = 12–19). (G) Homeostatic model assessment for insulin resistance (HOMA-IR) at 16 weeks, calculated as 5-h fasting glucose (mmol/l) × 5-h fasting insulin (ng/ml)/22.5 (n = 11–18). (H, I, and L) Fasting (16 h) and refed (6 h) free fatty acid (FFA), triacylglycerol (TAG), and leptin levels in 10-week-old CD-fed WT and *Adig*^*−/−*^ male mice (n = 4–6). (J and K) Random fed plasma FFA and TAG concentrations in WT and *Adig*^*−/−*^ mice fed a HFD (24 weeks) (n = 12–19). Data are expressed as mean ± SEM and were analyzed by 2-way ANOVA (A, C, and E; p value obtained from the interaction of genotype and diet), 1-way ANOVA with Bonferroni multiple-comparison post hoc testing (B, D, F–I, and L), or two-tailed Student’s t tests (J and K). ^∗^p < 0.05, ^∗∗^p < 0.01, ^∗∗∗^p < 0.001, ^∗∗∗∗^p < 0.0001.

Next, glucose tolerance and insulin response/sensitivity were evaluated by performing an intraperitoneal glucose tolerance test (ipGTT) at 16 weeks and an intraperitoneal insulin tolerance test (ipITT) at 19 weeks in mice fed CD or HFD. The glucose and insulin tolerance were similar between WT and *Adig*^−/−^ mice when fed a CD, but the expected deterioration in glucose and insulin tolerance in response to HF feeding was significantly ameliorated in the *Adig*^−/−^ group in keeping with their reduced weight gain and fat mass (Figures 2A–2D). Plasma insulin levels were significantly higher in the WT group when fed a HFD, remaining significantly lower in the *Adig*^−/−^ mice at the end of the study (Figures 2E and 2F). Homeostasis modeling assessment (HOMA-IR) values suggested that *Adig*^−/−^ mice were less insulin resistant than WT mice when fed a HFD for 16 weeks (Figure 2G). Fasting (16 h) and refed (6 h) free fatty acid and triacylglycerol concentrations were similar in CD-fed mice (Figures 2H and 2I), and circulating levels were also similar in HFD-fed (for 24 weeks) mice (Figures 2J and 2K). Plasma leptin concentrations in the fasting and refed mice rose as expected in response to feeding in both groups (Figure 2L).

### Impact of *Adig* deletion on leptin concentration, expression, and secretion from adipose explants

**Figure 3:**
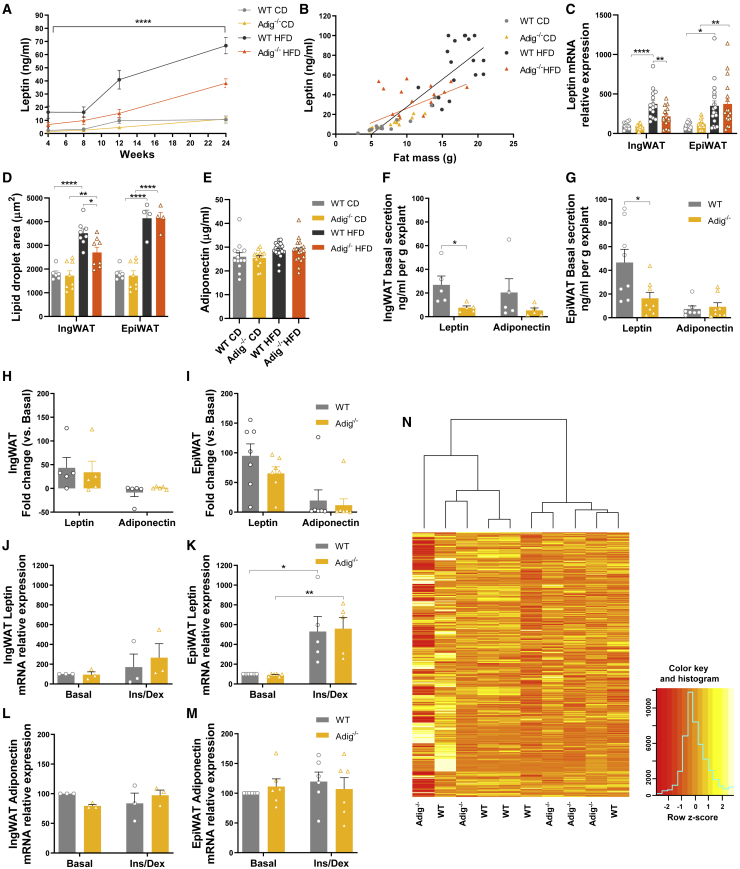
Impact of *Adig* deletion on leptin concentration, expression, and secretion from adipose explants (A) Random fed plasma leptin concentrations in samples from WT and *Adig*^−/−^ male mice fed a CD or HFD for 24 weeks (n = 13–18). (B) Correlation of leptin concentrations with fat mass at a 24-week time point (n = 14–17, regressions lines for the WT [black] and *Adig*^−/−^ mice [orange] are included). (C) *Leptin* mRNA expression in IngWAT and EpiWAT samples taken after 24 weeks on a CD or HFD relative to WT (set at 100) (n = 11–19). (D) Lipid droplet size analysis in IngWAT and EpiWAT samples obtained from mice after 24 weeks on a CD or HFD (n = 4–8). (E) Random fed plasma concentrations of adiponectin at the final time point (n = 13–19). (F–I) Leptin and adiponectin secretion from WT and *Adig*^−/−^ IngWAT and EpiWAT explants (from chow fed mice) following a 24-h incubation in standard medium or with added insulin (7 nM) and dexamethasone (25 nM) (secretion expressed as fold change) (n = 5–8). (J–M) Leptin and adiponectin mRNA expression in explants expressed relative to the basal WT sample (set at 100) (n = 3–6). (N) Heatmap showing the (standardized) RNA-seq counts per feature in the samples WT and *Adig*^*−/−*^ in IngWAT after 24 weeks of CD feeding (n = 5 per genotype). Data are expressed as mean ± SEM and were analyzed by 2-way ANOVA (A; p value obtained from the interaction of genotype and diet), 1-way ANOVA with Bonferroni multiple-comparison post hoc testing (C–E and J–M), or two-tailed Student’s t tests (F–I) . ^∗^p < 0.05, ^∗∗^p < 0.01, ^∗∗∗^p < 0.001, ^∗∗∗∗^p < 0.0001.

Plasma leptin concentrations were similar in chow-fed mice and increased significantly in response to a HFD in the WT group, as expected (Figure 3A). The leptin increase in HFD-fed *Adig*^−/−^ mice was somewhat attenuated (Figure 3A). In order to ascertain whether or not the relationship between leptin and fat mass (Figure 3B) was perturbed in the *Adig*^−/−^ mice, we generated a linear regression model including diet, fat mass, and genotype. The p value for the fat mass by genotype interaction was 0.005, and the association was clearly stronger in the WT mice, as shown by estimates of the difference in leptin concentration per gram of fat mass (WT: 3.88, 95% confidence interval [CI] = 2.61 to 5.15, p < 0.001; *Adig*^−/−^: 1.31, 95% CI = −0.41 to 3.02, p = 0.13). This difference appeared to largely be driven by the tendency for leptin concentrations to be lower in the HFD-fed *Adig*^−/−^ mice (Figure S2A), whereas leptin was very similar in CD-fed mice (Figure S2B). Leptin mRNA increased in HF-fed mice in EpiWAT but did so to a significantly reduced extent in IngWAT in *Adig*^−/−^ mice (Figure 3C). These changes in leptin expression corresponded with similar differences in lipid droplet area (Figure 3D), a measure that effectively represents cell size as well. However, when plotted relative to the fat mass of either the IngWAT or EpiWAT, leptin mRNA was similar in both groups (Figures S2C and S2D), suggesting that *Adig* might be affecting leptin secretion rather than mRNA expression. In contrast to the differences in leptin concentrations, plasma adiponectin concentrations were similar in both groups when fed a CD or HFD (Figure 3E).

Next, we sought to assess adipocyte leptin secretion directly by comparing leptin concentrations in the medium of WAT explants, both in the basal (unstimulated) state and following incubation with insulin and dexamethasone for 24 h. *Ex vivo* samples were derived from both IngWAT (subcutaneous) and EpiWAT (visceral) fat pads of chow-fed WT and *Adig*^*−/−*^ mice of similar weights. We also evaluated adiponectin expression and secretion as it is predominantly expressed and secreted from WAT as well, but its levels *in vivo* were not changed by *Adig* deletion (Figure 3E). The data suggested that basal leptin secretion was significantly reduced in the *Adig*^−/−^ IngWAT and EpiWAT explants, whereas adiponectin secretion did not differ from that observed in WT explants (Figures 3F and 3G). Note that leptin secretion is ∼2-fold greater from EpiWAT explants than it is from IngWAT explants. This result could relate to the substantial differences in manual handling required to isolate epididymal explants compared to inguinal explants that have to be dissected free from the overlying skin. In response to stimulation with insulin and dexamethasone, which have previously been shown to induce leptin secretion in AT explants (Lee et al., 2007), the relative increase in leptin secretion from *Adig*^*−/−*^ IngWAT and EpiWAT was similar to that seen in WT samples, whereas no change in adiponectin secretion was observed in response to these stimuli (Figures 3H and 3I). These changes are not a result of differences in leptin mRNA expression, as this was similar in basal and insulin/dexamethasone-treated samples (Figures 3J–3M), suggesting again that Adig has an effect on leptin secretion or possibly its translation. In keeping with this suggestion, RNA sequencing (RNA-seq) analysis of IngWAT samples suggests that *Adig* deletion does not have a significant impact on gene expression in chow-fed mice (Figure 3N).

### Leptin expression and secretion in cultured adipocytes

Although leptin expression in cultured adipocytes is reportedly very low (Zeigerer et al., 2008), we next sought to study the impact of *Adig* KD in cultured adipocytes. In both 3T3-L1 cells, in which *Adig* expression was effectively knocked down using small interfering RNA (siRNA), and in primary pre-adipocytes derived from the stromovascular fraction (SVF) of WT and *Adig*^−/−^ mice, an analysis of lipid accumulation and adipocyte gene expression suggested that adipogenesis was significantly impaired in *Adig*-deficient cells, making it difficult to discern an independent effect on leptin expression or secretion (See Supplemental information related to Figure S3 for details).

### Impact of *Adig* deletion in hyperphagic *Ob/Ob* mice

**Figure 4:**
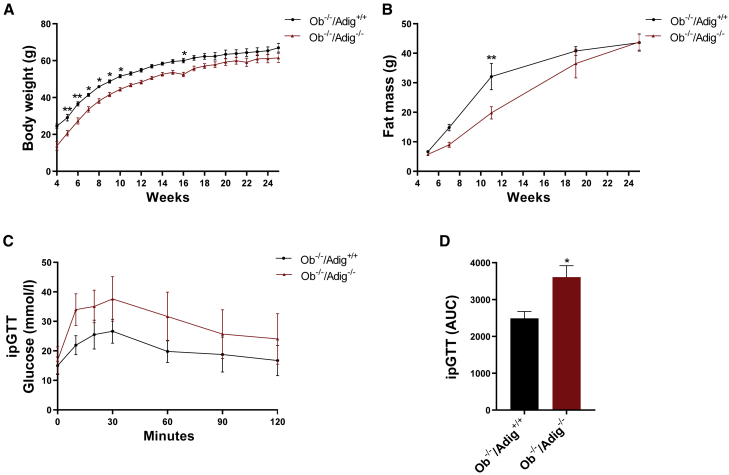
Impact of *Adig* deletion in hyperphagic *Ob/Ob* mice (A and B) Weekly body weight (n = 8–11) (A) and fat mass (n = 4–10) (B) measurements in *Ob*^*−/−*^*/Adig*^*+/+*^ and *Ob*^*−/−*^*/Adig*^*−/−*^ male mice over 25 weeks on a chow diet. (C and D) ipGTTs and the corresponding AUC analysis (n = 8–10) performed at 21 weeks of age. Data are expressed as mean ± SEM and were analyzed by 2-way ANOVA with Bonferroni multiple-comparison post hoc testing (A–C) or a two-tailed Student’s t test (D).^∗^p < 0.05, ^∗∗^p < 0.01.

Modest reductions in fat mass have been reported in several mouse models of partial lipodystrophy in which the genetic defects are known to affect adipocyte differentiation (Savage, 2009). In some of these models, crossing the mice with hyperphagic leptin-deficient *Ob/Ob* mice led to an obviously lipodystrophic diabetic phenotype (Medina-Gomez et al., 2007; Zhou et al., 2015); so, we next crossed the *Adig*^−/−^ mice with *Ob/Ob* mice. Clearly, in this setting, the impact of *Adig* deletion on leptin secretion is not examinable; thus, this leptin-deficient model can be used only to assess the impact of *Adig* deficiency on the response to extreme hyperphagia. Surprisingly, given the differences in weight gain in HFD-fed WT and *Adig*^*−/−*^ mice (Figure 1C) in the double knockout (*Adig* and *Lep*) *Ob*^*−/−*^*/Adig*^*−/−*^ mice, body weight and fat mass were rather modestly reduced compared to that of *Ob*^*−/−*^*/Adig*^*+/+*^ mice (Figures 4A and 4B). However, glucose tolerance was now significantly worse in the *Ob*^*−/−*^*/Adig*^*−/−*^mice than in the standard *Ob*^*−/−*^*/Adig*^*+/+*^ mouse line (Figures 4C and 4D).

## Discussion

*Adig* is a small membrane protein most highly expressed in AT in mice. It is predicted to be a type I protein with its amino-terminus extending into the lumen if it localizes to the endoplasmic reticulum, although this still requires formal experimental validation. Its principle expression in AT is consistent with the fact that the gene itself is present only in species with clearly defined white adipocytes, i.e., Sauria and Mammalia (see Supplemental information; Figure S4; Kim et al., 2005). Initial studies on it showed that its expression was induced during adipogenic differentiation of 3T3-L1 adipocytes and that, *in vivo*, its mRNA was detectable in the adipocyte rather than the SVF of AT samples (Hong et al., 2005; Kim et al., 2005). Two groups initially studied the impact of *Adig* KD on adipogenesis, with one reporting that *Adig* deficiency did (Hong et al., 2005) and the other (Ren et al., 2016b) that it did not impair adipogenesis. Our data suggest that these observations can be reconciled by the timing of the initiation of *Adig* KD, as we clearly show that early KD significantly impairs lipid accumulation in 3T3-L1 adipocytes as well as expression of several typical adipocyte genes (Figures S3A–S3D), whereas late (day 5 onward) KD in 3T3-L1 cells does not clearly impair differentiation (Figures S3E–S3H). This observation is supported by the similarly impaired adipogenic capacity of *Adig*-deficient primary SVF-derived pre-adipocytes. Despite this consistent and substantial impairment in the differentiation potential of these rodent cell types, *Adig*^*−/−*^ mice fed a normal CD have morphologically normal white fat depots for up to 24 weeks. Furthermore, RNA-seq analysis of gene expression in WAT samples showed that the expression of typical WAT genes was similar in *Adig* null and WT mice, as were circulating levels of adipocyte-secreted proteins (leptin and adiponectin) and free fatty acids. WAT function also appeared to be normal in terms of its impact on glucose and insulin tolerance and fasting/refeeding transitions, although more detailed analyses have yet to be performed.

However, when exposed to a HFD, weight gain in *Adig*^*−/−*^ mice is significantly attenuated, to the extent that the mice largely follow growth curves of chow-fed mice, only diverging at ∼20 weeks after the initiation of high fat feeding. This result does not appear to be a consequence of reduced food intake. EE analysis suggests that it is increased in the *Adig*^*−/−*^ mice, although this finding could reflect, at least in part, the fact that the mice have less insulating WAT at the age at which EE was evaluated and were housed at a temperature below thermoneutrality. So, we cannot formally establish whether the primary defect was due to reduced fat mass accrual with a secondary increase in EE or vice versa.

In addition to being an element required for optimal adipogenesis, at least in cultured cells, *Adig* is also of considerable scientific interest, as the human GWAS study suggested that a locus near the *ADIG* gene influenced BMI-adjusted plasma leptin levels (Kilpeläinen et al., 2016). Although leptin concentrations clearly correlate with fat mass in humans and mice, exactly how leptin expression, synthesis, and secretion are regulated within individual adipocytes remains poorly understood (Friedman, 2016 ). The human GWAS data suggested that *ADIG* might be involved in this process, so we were keen to explore this aspect in more detail.

Our data suggest that *Adig* is not required for leptin synthesis or secretion, as leptin levels were similar in chow-fed WT and *Adig*^*−/−*^ mice. Furthermore, in WAT explants from both subcutaneous inguinal and visceral epididymal fat pads, the capacity of insulin and dexamethasone to induce leptin secretion was unimpaired, and leptin changes in fasting and refed mice were similar in both groups. However, we did observe altered fat-mass-adjusted leptin levels in *Adig*^*−/−*^ mice and a significant reduction in leptin concentrations in the media in which explants from both depots were incubated. This does not appear to be secondary to a “general impairment” in adipokine secretion, as adiponectin secretion from *Adig*^*−/−*^ explants was similar to that of WT. As leptin mRNA expression was also similar in *Adig*^*−/−*^ and control explants, the data suggest that Adig deficiency may impact the synthesis and/or secretion of leptin, although how this is mediated remains unclear.

We elected to cross the *Adig*^*−/−*^ mouse line with leptin-deficient *Ob/Ob* mice for two reasons. First, we wanted to evaluate the impact of this dual perturbation on glucose metabolism, as we conventionally expected impaired adipogenesis to translate into a “lipodystrophic insulin-resistant” phenotype. As this was not apparent in the high-fat feeding paradigm in which glucose and insulin tolerance remained consistent with the lower body weight of the HFD-fed *Adig*^*−/−*^ mice, we wanted to subject the mice to a more substantial overfeeding stress. This approach was needed to reveal the lipodystrophic phenotype of previous mouse models of partial lipodystrophy, such as the P467L PPARγ knockin mouse model (Gray et al., 2006) and the *Cidec* null model (Zhou et al., 2015). Interestingly, when the *Adig*^*−/−*^ mouse line was crossed onto an *Ob/Ob* background, the reduction in fat mass accrual was rather modest, although the mice did then manifest impaired glucose tolerance. Second, we performed this cross to assess the impact of Adig deficiency in a setting in which leptin was not present. In this regard, the data suggest that Adig has an impact on fat mass independent of leptin. In our view, it would also be of interest to cross the *Adig* null mice with leptin-receptor-deficient *Db/Db* mice, as this would enable one to assess the impact of Adig deficiency on leptin concentrations in the setting of extreme hyperleptinaemia.

Our studies were limited by the lack of a reliable antibody to mouse Adig, so we were not able to confirm the deletion of Adig at the protein level, although the degree of mRNA KD does suggest that the knockout strategy was highly effective. More importantly, the lack of a reliable antibody has hindered efforts to identify endogenous protein-protein interaction partners for Adig *in vivo*, so we have yet to reveal exactly how Adig deficiency affects differentiation of 3T3-L1 adipocytes and leptin secretion. The remote similarity to membrane regulatory proteins phospholamban and sarcolipin might provide some clues for future research (see Supplemental information; Figure S4).

In summary, our data clearly suggest that Adig deficiency impairs adipocyte differentiation in cultured cells and that, *in vivo*, it attenuates fat and weight gain in mice. In leptin-deficient *Ob/Ob* mice, Adig deficiency only modestly impairs fat mass accrual but does exacerbate glucose intolerance. The data also suggest that Adig probably does have an additional impact on leptin secretion, although we have yet to establish exactly what *Adig* does in either context.

## STAR★Methods

### Key Resources Table

**Table:** 

REAGENT or RESOURCE	SOURCE	IDENTIFIER
**Biological samples**		

*Adig*^tm1.1(KOMP)Vlcg^ mice tissues	In this study (University of Cambridge)	NA

**Chemicals, peptides, and recombinant proteins**		

Tri-Reagent	Sigma-Aldrich	Cat# T9424
Chloroform	Honeywell	Cat# C2432
Ethanol, puriss. p.a., absolute, ≥ 99.8% (GC)	Sigma-Aldrich	Cat# 32221-M
Dulbecco’s Minimum Essential Medium (DMEM)	Sigma-Aldrich	Cat# D6546
Dulbecco’s Minimum Essential Medium (DMEM/F12)	Sigma-Aldrich	Cat# D9785
Medium 199, Earle’s Salts	Thermo Fisher Scientific	Cat# 11150059
D-PBS	Sigma-Aldrich	Cat# D8537
Hanks’ Balanced Salt Solution	Sigma-Aldrich	Cat# H9269
L-Glutamine	Sigma-Aldrich	Cat# G7513
Penicillin-Streptomycin	Sigma-Aldrich	Cat# P0781
MEM Non-essential Amino Acid	Sigma-Aldrich	Cat# M7145
Sodium pyruvate	Sigma-Aldrich	Cat# S8636
Fetal Bovine Serum	PAN-Biotech	Cat# P30-3602
Newborn Calf Serum	Sigma-Aldrich	Cat# N4637-500M
Insulin (Actrapid)	Novo Nordisk	Cat# 041-7642
3-isobutyl-1-methylxanthine, IBMX	Sigma-Aldrich	Cat# I7018
Dexamethasone	Sigma-Aldrich	Cat# D4902
Rosiglitazone	Sigma-Aldrich	Cat# R2408
AdipoRed Assay Reagent	Lonza	Cat# PT-7009
Lipofectamine RNAiMAX	Invitrogen	Cat# 13778-150
Taqman MasterMix	Applied Biosystems	Cat# 4304437
dNTPs	Promega	Cat# U151B
Random primers	Promega	Cat# C1181
RNasin Plus Ribonuclease inhibitor	Promega	Cat# N2611
MMLV Reverse Transcriptase	Promega	Cat# M1701
Collagenase Type II from Clostridium histolyticum	Sigma-Aldrich	Cat# C6885
Collagenase Type I from Clostridium histolyticum	Sigma-Aldrich	Cat# SCR103
Bovine Serum Albumin	Sigma-Aldrich	Cat# A6003
Formalin solution neutral buffered 10%	Sigma-Aldrich	Cat# HT501128
Haematoxylin (Mayer)	Pioneer Research Chemicals	Cat# PRC/R/42
Eosin (1٪ aqueous)	Pioneer Research Chemicals	Cat# PRC/66/1
Paramat Gurr Paraffin Wax	VWR	Cat# 361147B
Xylene	Thermo Fisher Scientific	Cat# 12632916

**Critical commercial assays**		

RNeasy Mini Kit	QIAGEN	Cat# 74106
Qiashredder	QIAGEN	Cat# 79656
Mouse Insulin and Leptin Assay	MesoScale Discovery	Cat# K15124C-3
Mouse Adiponectin Assay	MesoScale Discovery	Cat# K152BYC-2
Free Fatty Acids Assay	Roche	Cat# 11383175001
Triglycerides Assay	Siemens Healthcare	Cat# DF69A
Illumina® TruSeq® Stranded mRNA	Illumina	Cat# 20020594

**Experimental models: cell lines**		

3T3-L1	ATCC	Cat# CRL-3242 RRID: CVCL_0A20
Mouse primary adipocytes	C57BL/6J	In house
Ear mesenchymal Stem Cells	C57BL/6J	In house

**Experimental models: organisms/strains**		

M. musculus C56Bl/6N *Adig*^tm1.1(KOMP)Vlcg^ mice strain	KOMP	Cat# 046516-UCD; RRID:MMRRC_046516-UCD
M. musculus C56Bl/6J *Adig*^tm1.1(KOMP)Vlcg^ mice strain	In this study (University of Cambridge)	NA
M. musculus C56Bl/6J B6.Cg-Lepob/J mice strain	Jackson laboratories	Cat# JAX:000632; RRID:IMSR_JAX:000632
M. musculus C56Bl/6J *Adig*^tm1.1(KOMP)Vlcg^ + B6.Cg-Lepob/J mice strain	In this study (University of Cambridge)	NA

**Oligonucleotides**		

See Table S1 for oligonucleotide information		NA

**Software and algorithms**		

GraphPad PRISM 8.4.0 (671)	1992-2020 GraphPad Software, LLC	RRID: SCR_002798
HALO	Indica Labs	NA
Blast and PsiBlast	NCBI database	https://www.ncbi.nlm.nih.gov
MPI Bioinformatics Toolkit	MPI	https://toolkit.tuebingen.mpg.de/
PolyPhobius	NA	https://phobius.sbc.su.se/poly.html
Jalview 2.11.1.0	NA	www.jalview.org
T-coffee	NA	http://tcoffee.crg.cat/
Cutadapt 2.10	Cutadapt	https://cutadapt.readthedocs.io/en/stable/
STAR 2.7	NA	NA
Feature Counts 1.6.2	Subread	http://subread.sourceforge.net/
R package 1.26.0	NA	https://www.r-project.org/
heatmap.2 and gplots2 v. 3.03 8 (R package 1.26.0)	NA	https://www.r-project.org/

**Deposited data**		

RNaseq raw and analyzed data	In this study	GEO: GSE158005

**Other**		

Chow diet (mouse studies)	Safe Diets	Cat# R105-25
45% High Fat Diet (mouse studies)	Research Diets	Cat# D12451i
QuantStudio 7 Flex Real time PCR system	Thermo Fisher Scientific	NA
Nanodrop 2000	Thermo Fisher Scientific	NA
FastPrep-24	MP Biomedical	Cat# 116004500
AlphaTrack2 Glucometer	Abbot Laboratories	Cat# CFMU305-H0201
AlphaTrack2 strips	Zoetis	Cat# 71681-01
Lysing Matrix D, 2 mL Tube	MP Biomedical	Cat# 116913100
Sterile Cell strainer (100 μm nylon mesh)	Fisherbrand	Cat# 22363549
Minispec LF series (TD-NMR)	Bruker	Cat# LF50
Meta-Traxe (SMS) System (Indirect calorimetry)	Custom build	NA
Microtome	Leica	Cat#RM2255
Axio Scan Z1 slidescanner	Zeiss	NA
HistoStar embedding workstation	Thermo Fisher Scientific	NA
M1000 Pro Plate Reader	Tecan	NA
2100 Bioanalyzer Instrument	Agilent Technologies	NA
HiSeq 4000 Sequencing System	Illumina	NA

### Resource availability

#### Lead contact

Further information and requests for resources and reagents should be directed to and will be fulfilled by the Lead Contact, Prof David B Savage (dbs23@medschl.cam.ac.uk).

#### Materials availability

The study did not generate new unique materials or reagents.

#### Data and code availability

The accession number for the datasets reported in this paper is: GEO: GSE158005.

### Experimental model and subject details

Mouse lines used in this study are available to the Knockout Mouse Project (KOMP), (Cat# 046516-UCD RRID:MMRRC_046516-UCD) or from Jackson laboratories (Cat# JAX:000632 RRID:IMSR_JAX:000632).

*Adig*^*−/−*^ mouse sperm (defined as *Adig*^*tm1.1(KOMP)Vlcg*^*)* was purchased from KOMP, UC Davis on a C57BL/6N background. After *in vitro* fertilization, the resultant *Adig*^*+/−*^ mice were backcrossed to wild-type C57BL/6J mice. Using Marker-Assisted Accelerated Backcrossing (MAX_BAX®, Charles River), 5 backcrosses were needed to achieve a C57BL/6J pure background. *Adig*^*−/−*^ and the WT littermates were then bred in-house by crossing *Adig* heterozygotes. B6.Cg-Lepob/J (*Ob*^*+/−*^) were purchased from the Jackson Laboratories and crossed to the C57BL/6J Adig heterozygous mice to obtain *Ob*^*−/−*^*/Adig*^*+/+*^ and *Ob*^*−/−*^*/Adig*^*−/−*^ littermates.

Genotyping was done by PCR using the primers described in the Table S1. Mice were maintained in ventilated cages with group housing (2-4 per cage), unless specified otherwise for indirect calorimetry and food intake experiments, on a 12 h light/12 h dark cycle (lights on 06:00–18:00), in a temperature-controlled (20-24°C) facility, with *ad libitum* access to food and water. During the experimental protocol, all mice were fed either *ad libitum* or fasted as stated otherwise prior to some tests.

This research was regulated under the Animals (Scientific Procedures) Act 1986 Amendment Regulations 2012 following ethical review by the University of Cambridge Animal Welfare and Ethical Review Body (AWERB). Male mice were used in all the protocols of this study, whereas females were used for the high fat diet paradigm study.

### Method details

#### High fat diet study

5 week-old WT and *Adig*^*−/−*^ littermate male and female mice were fed either a chow (R105-25, Safe Diets) or a 45% high fat diet (D12451i, Research Diets) for a period of 24 weeks. All mice were weighed weekly and body composition determined every 3-4 weeks by Time-Domain Nuclear Magnetic Resonance (TD-NMR) using a Minispec Live Mouse Analyzer (LF50, Bruker). Random fed tail vein blood samples were collected every 4 weeks into heparinized micro blood tubes (01605-00, Hawksley), centrifuged at 13,000 x g for 4 min, and plasma was collected for leptin, adiponectin, insulin, free fatty acids and triglyceride measurements. At the same time, mouse glucose levels were measured from approximately 2 μl blood drops using a glucometer (AlphaTrak2; Abbot Laboratories) and glucose strips (AlphaTrak2 test 2 strips, Abbot Laboratories, Zoetis). At the end of the study, TD-NMR and blood collection was performed prior to sacrifice; with tissues harvested, weighed and stored as indicated until further processing.

#### Overnight fasting and refeeding study

10 week-old WT and *Adig*^*−/−*^ littermate male mice fed a chow diet, were fasted for a 16-hour period (4.00 pm-8.00 am), followed by 6 hours of *ad libitum* refeeding with CD. Fasting and refeeding tail vein blood samples were collected in the early morning as previously described for free fatty acids, triacylglycerol and leptin measurements.

#### Ob/Ob cross study

All mice were maintained as described previously and with *ad libitum* access to a chow diet and water, or fasted as stated otherwise prior to some tests, for a period of 24 weeks. Blood tail glucose was measured weekly and ketone bodies were assessed in urine when glucose was higher than 30 mmol/l, as a control for diabetes. Body weight and composition, plasma measurements and tissue harvesting were performed as described for the HFD study.

#### Glucose and insulin tolerance tests

Intraperitoneal glucose (ipGTT) and intraperitoneal insulin tolerance tests (ipITT) were performed in chow or high fat diet fed mice after 16 and 19 weeks respectively. Following a 4 hour fast (starting at 8.00 am) mice were single housed and left to habituate for 2 further hours. For both tests, basal blood glucose from the tail vein was measured followed by an intraperitoneal injection of either 1 g/kg glucose in the ipGTT or 0.75 U/kg insulin in the ipITT. Blood glucose measurements were determined at 10, 20, 30, 30, 90 and 120 minutes after injection. In the *Ob/Ob* cross study, ipGTT was performed at weeks 21 using a bolus of 1 g/kg glucose.

#### Indirect calorimetry and food intake measurements

Male mice were single-housed for a week prior to indirect calorimetry analyses (21 weeks CD or HFD) in a custom built calorimetry system (the Meta-Traxe (SMS) System) for up to 48 hours. Carbon dioxide (CO_2_) and oxygen (O_2_) concentrations and the incoming air supply were determined every 11 minutes for each chamber/mouse. Energy expenditure was calculated using the modified Weir equation [EE J/min = 15.818xVO_2_ (ml/min) + 5.176^∗^VCO_2_ (ml/min)]. Metabolic flexibility was assessed by measuring the amplitude of respiratory exchange ratio (RER) from mice in free living calorimetry chambers (Virtue et al., 2018).

Following the indirect calorimetry study, mice were returned to individual clean ventilated cages with a surgical cage liner in the otherwise empty base. ‘Environmental enrichment toys’ were supplied. Mouse and food weight were recorded daily for a 2 week period.

#### Adipose tissue and liver histological analysis

At the time of sacrifice, various adipose depots and liver tissue were dissected with a small piece fixed in 10% formalin (HT501128, Sigma) for 5-7 days and kept in 70% ethanol until paraffin processing. Tissues were embedded in paraffin overnight for further cutting. 4 μm sections were obtained using a Leica RM2255 microtome and mounted on slides. The tissue sections were processed for hematoxylin/eosin (PRC/R/42, PRC/66/1, Pioneer Research Chemical) staining and imaged using a Axio Scan Z1 slide scanner (Zeiss). Lipid droplet area was quantified automatically using Halo software (Indica Labs).

#### RNA isolation and gene expression analysis

At the end of the study, tissues were harvested and immediately snap frozen in liquid nitrogen and stored at −80°C until further analysis. For RNA isolation, approximately 30-50 mg of tissue was placed in Lysing Matrix D tubes and homogenized in 800 μl TRI Reagent (T9424, Sigma) using the Fastprep-24 Homogenizer for 30 s at 4-6 m/s (MP Biomedical). The resultant homogenate was transferred to an RNase free tube and 200 μl chloroform (Sigma) added. The samples were vortexed and centrifuged at 13,000 rpm for 15 min at 4°C. The upper phase was then transferred to an RNase free tube and mixed with an equal volume of 70% ethanol before loading onto RNA isolation spin columns. RNA was then extracted using a RNeasy Mini Kit (74106, QIAGEN) isolation kit following the manufacturer’s instructions.

For cell RNA isolation, RLT lysis buffer and QIAshredder columns (79656, QIAGEN) along with the RNeasy Mini isolation kit was used.

RNA concentration and quality were determined using a Nanodrop analyzer. 400 ng of total RNA was converted to cDNA using MMLV Reverse Transcriptase with random primers (Promega). Quantitative RT-PCR was carried out with either TaqMan Universal PCR Master Mix or SYBR Green PCR master mix on the QuantStudio 7 Flex Real time PCR system (Applied Biosystems) in a 10 μl volume using 2 μl cDNA, 1:10 diluted. All reactions were carried out in either duplicate or triplicate and Ct values were obtained. Relative differences in the gene expression were normalized to expression levels of housekeeping genes, Cyclophillin A or B2M for cell analysis and to HPRT, B2M and 36b4 geometrical mean for mouse data, using the standard curve method. Primer sequences are shown in Table S1.

#### Transcriptome mRNA sequencing (RNaseq)

RNA isolated from IngWAT, as described above, was used for RNaseq analysis. RNA quality was analyzed using a bioanalyser (Agilent technologies) and samples with a RIN number higher than 8 were used. 200 ng mRNA were sampled for the preparation of an mRNA library was prepared using the reagents provided in the Illumina® TruSeq® Stranded mRNA library prep (20020594, Illumina) workflow. Briefly, messenger RNA was enriched from total RNA before reverse transcription. Adenylation and barcode ligation was performed after the synthesis of double stranded cDNA. Ligated libraries were enriched with a limited amplification. Libraries from individual samples were combined at equal molar concentration of DNA, before loading onto one lane of either an Illumina HiSeq™ 4000 (Illumina) instrument. Sequencing was performed at the Genomics Core, Cancer Research UK, Cambridge Institute, Cambridge.

The reads obtained from IngWAT RNaseq were adapted and quality trimmed using Cutadapt v. 2.10 (parameter -q was set to 10) and subsequently aligned to the reference genome (*Mus musculus*, GRCm38) using STAR 2.7. The number of reads per reference feature was computed with Feature Counts 1.6.2. The differential gene expression was estimated relying on the R package (R core Team, 2019. R: A language and environment for statistical computing. R Foundation for Statistical Computing, Vienna, Austria) DESeq2 1.26.0 (Love et al., 2014). Of the sequenced reads, an average of 3% was filtered out in the trimming step. Of the remaining reads, 75% were successfully aligned to the reference. Finally, an average of the 79% of the genes were correctly assigned to the respective features. The heatmap was produced using the function heatmap.2 of the R1 package gplots2 3.03.

#### Plasma and media biochemical analyses

Plasma samples obtained during the *in vivo* study or media samples collected from incubated explants were stored at −80°C for the insulin, leptin and adiponectin assays. Mouse insulin and leptin were measured simultaneously using a 2-plex Mouse Metabolic immunoassay kit (K15124C-3, MSD) while adiponectin (K152BYC-2, MSD) was analyzed individually using the Meso Scale Discovery Kit (Rockville, MD, USA). The assays were performed according to the manufacturer’s instructions and using recombinant human insulin and mouse leptin and adiponectin as calibrators. FFA were analyzed using the Free Fatty Acid Kit (half-micro test) (11383175001, Roche) and TAG was measured using and enzymatic assay (DF69A, Siemens Healthcare). All sample measurements were performed by the MRC MDU Mouse Biochemistry Laboratory.

### *In vitro* and *ex vivo* adipogenesis studies

#### Adipose tissue explants

Inguinal (subcutaneous) and epididymal (visceral) adipose tissue was harvested from 10-12 weeks old male mice fed a chow diet and placed in Hanks’ Balanced Salt Solution (HBSS, H9269, Sigma) and kept on ice. Tissue was cut into 1-2 mm fragments and approximately 100 mg incubated in a 12-well plate with M199 media ± 7 nM insulin (Actrapid, Novo Nordisk) and 25 nM dexamethasone (D4902, Sigma). After 24 hour incubation in basal or insulin plus dexamethasone treated conditions, media was collected, spun down at 5,000 g and stored at −80°C until further leptin and adiponectin measurements as described above. The explant tissues were weighed and snap frozen for RNA processing and analysis.

#### 3T3-L1 adipocytes

Mouse 3T3-L1 cells were obtained from ATCC and maintained in Dulbecco’s Minimum Essential Medium (D6546, Sigma) supplemented with 10% (vol/vol) Newborn calf Serum (NCS, P30-3602, Pan-Biotech), 2 mM L-glutamine, penicillin/streptomycin, 1% Sodium Pyruvate, 1% Non-Essential Amino Acids at 37C in a humidified atmosphere of 5% CO_2_. 3T3-L1 preadipocytes seeded onto 12-well plates (Corning) were induced to differentiate into adipocytes 2 days after reaching confluence (Day −2) in maintenance media (DMEM 10% NCS). On day 0, media was changed to DMEM 10% Fetal bovine Serum (FBS, P30-3602, Pan-Biotech) plus the differentiation cocktail which included 1 μM insulin (Actrapid, Novo Nordisk), 500 μM 3-isobutyl-1-methylxanthine, IBMX (I7018, Sigma) and 1 μM dexamethasone (D4902, Sigma) for 2 days. Next, media was replaced with DMEM 10% FBS plus 1 μM insulin followed by media changes every second day. In parallel, cells were transfected with one of two different siRNAs (See Table S1) at 30 nM for *Adig* (siAdig1: Silencer Select siRNA S110859, Invitrogen and siAdig2: J-041009-10-0002, Dharmacon) or with 30 nM scrambled siRNA from Invitrogen or Dharmacon (for each of the siAdig analyzed) using Lipofectamine RNAi MAX (13778-150, Invitrogen) according to the manufacturer’s instructions. siRNA transfections were initiated either on day 0 or day 5 of differentiation, depending on the experimental set up. On day 8 of differentiation, 48h accumulated media was collected for leptin and adiponectin quantification as described above. The cells were subsequently stained with 30 μl/well AdipoRed (PT-7009, LONZA) for 15 minutes at 37°C and the fluorescence read on a Tecan Sparks (M1000 Pro Plate Reader, Tecan) with excitation at 485 nm and emission at 572 nm. Following staining, the cells were lysed and processed for RNA and gene expression analysis as previously described.

#### Primary adipose tissue preadipocytes – Stromal Vascular Fraction differentiation

Inguinal adipose tissue was harvested from 10-12-week old male mice fed a chow diet and placed in Hanks’ Balanced Salt Solution (HBSS, H9269, Sigma) and kept on ice. Tissue was minced thoroughly and resuspended into 5 mL digestion solution (2.25% BSA (A6003, Sigma) and 10 mg Collagenase Type II (C6885, Sigma) in HBSS and incubated at 37°C with sustained 250 rpm shaking for 10-20 minutes, until the tissue was digested. The digestion mixture was then passed through a 100 μm cell strainer (352360, Falcon) into a fresh tube and incubated on ice for 10 minutes. Next, the digested material was centrifuged at 700 x g (at 4°C) for 10 min and the pellet was resuspended in 2ml red blood lysis buffer (Roche, B00003) for 2 minutes at room temperature and topped-up to 15 mL with DMEM 10% FBS prior to re-centrifugation. The cell pellet containing the stromal vascular fraction (SVF) was resuspended in 2 mL of medium (for each mouse) and seeded into 4 wells of a 24-well plate per mouse. 2 days after cells reached confluence, adipocyte differentiation was induced using the same protocol as was used for the 3T3-L1 cells (described above) in the absence or presence of Rosiglitazone (R2408, Sigma). Cells were stained with AdipoRed and harvested for RNA extraction on day 12 of differentiation as previously described.

#### Gene, protein, phylogeny and membrane topology (Bioinformatics)

Homology searches were performed with Blast and PsiBlast algorithms as implemented in the NCBI database (https://www.ncbi.nlm.nih.gov) and MPI Bioinformatics Toolkit (Zimmermann et al., 2018). Membrane structure and topology were predicted with PolyPhobius (Käll et al., 2005). The sequence alignments were performed using T-coffee (Di Tommaso et al., 2011) (http://tcoffee.crg.cat/) and analyzed in Jalview (www.jalview.org).

### Quantification and statistical analysis

Quantitative data is reported as mean ± SEM. As indicated in the figure legends, differences between means were assessed by two-tailed Student’s t tests or, 1-way ANOVA or 2-way ANOVA with Bonferroni multiple comparison post hoc testing using GraphPad Prism software (GraphPad 8.4.0 (671), San Diego). In order to evaluate the impact of *Adig* deletion on the relationship between fat mass and leptin concentration *in vivo*, we generated a linear regression model as outlined in the results section. Statistical significance was defined as p < 0.05 (^∗^p < 0.05, ^∗∗^p < 0.01, ^∗∗∗^p < 0.001, ^∗∗∗∗^p < 0.0001).

## Supporting citations

The following references appear in the supplemental information: Negi et al. (2015) and Shaikh et al. (2016).

## Supplemental information

Document S1. Figures S1–S4 and Table S1

Document S2. Article plus supplemental information
