# Supplementary material for: Phenotypic characterization of Adig null mice suggests roles for adipogenin in the regulation of fat mass accrual and leptin secretion
Source: Cell Rep. 2021 Mar 9;34(10):108810. doi: 10.1016/j.celrep.2021.108810 (PMC7966854; doi:10.1016/j.celrep.2021.108810)
Supplement: Document S1. Figures S1–S4 and Table S1 [file mmc1.pdf]

Cell Reports, Volume *34*

Supplemental information

Phenotypic characterization of *Adig* null mice suggests roles for adipogenin in the regulation of fat mass accrual and leptin secretion

Anna Alvarez-Guaita, Satish Patel, Koini Lim, Afreen Haider, Liang Dong, Olivia J. Conway, Marcella K.L. Ma, Davide Chiarugi, Vladimir Saudek, Stephen O'Rahilly, and David B. Savage

Figure S1. *Adig* impact on body weight (Related to Figure 1)

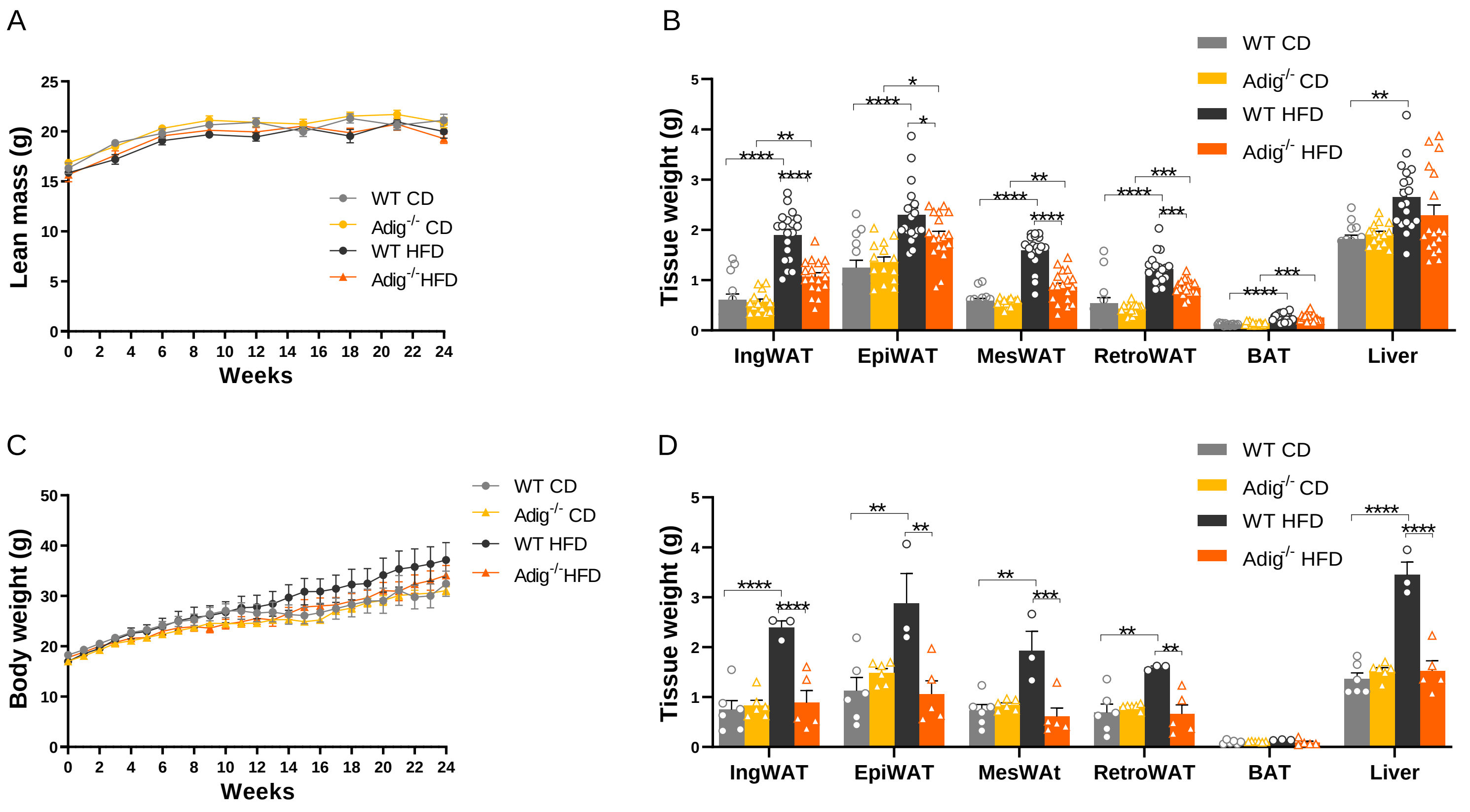

Figure S1. *Adig* impact on body weight (Related to Figure 1)

(A) Lean mass from WT and *Adig*$^{-/-}$ male mice (aged 5 weeks) fed a chow diet (CD) or high-fat diet (HFD) for 24 weeks (n=14-21). (B) Tissue weights measured at the end of the experiment (n= 14-19).

(C) Body weights recorded weekly from WT and *Adig*$^{-/-}$ female mice (aged 5 weeks) fed a CD or HFD for 24 weeks (n=6-12). (D) Tissue weights measured at the end of the experiment (n= 3-6).

Data is expressed as mean ± SEM and was analysed by 2-way ANOVA (A and C p value obtained by comparing genotypes and diets) or 1-way ANOVA (B and D compared to WT CD) with Bonferroni multiple comparison post-hoc testing. *p <0.05, **p < 0.01, ***p < 0.001, ****p < 0.0001. White adipose tissues - Inguinal (IngWAT), epididymal (EpiWAT), mesenteric (MesWAT), and retroperitoneal (RetroWAT). Brown adipose tissue (BAT).

Figure S2. Impact of Adig deletion on plasma leptin and leptin mRNA levels relative to fat mass (Related to Figure 3)

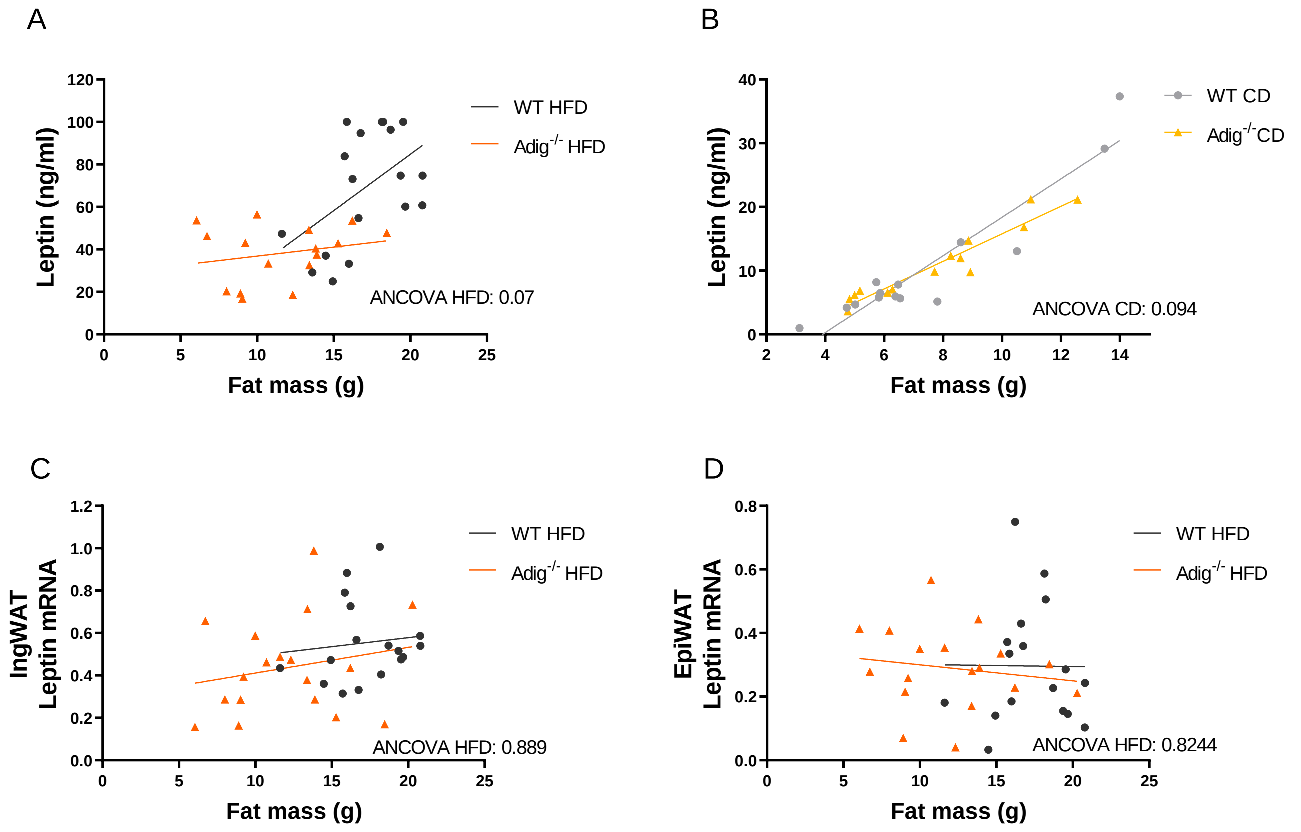

Figure S2. Impact of Adig deletion on plasma leptin and leptin mRNA levels relative to fat mass (Related to Figure 3)

(A-B) Plasma leptin and fat mas correlation at the 24 week time-point in WT and *Adig*$^{-/-}$ fed a HFD or CD (n= 14-18). (C-D) Leptin mRNA relative expression correlated to fat mass at the same time point in IngWAT and EpiWAT (n= 16-18).

Data is presented as scatter plot and ANCOVA shown as p value for slope difference between genotypes and diets.

Figure S3. Leptin expression and secretion in cultured adipocytes (Related to Figure 3)

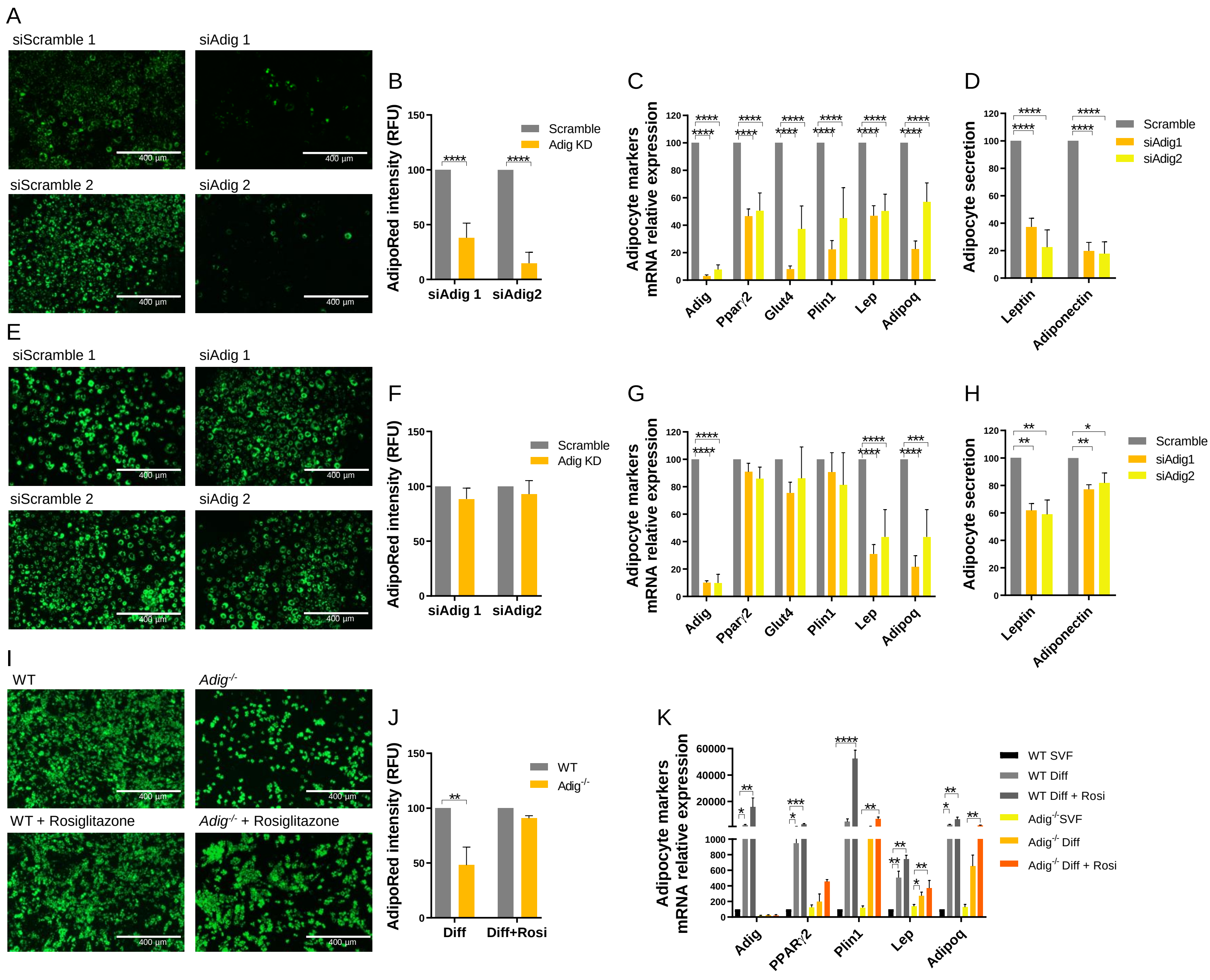

Figure S3. Leptin expression and secretion in cultured adipocytes (Related to Figure 3)

(A-C) *Adig* was knocked down in 3T3-L1 preadipocytes from the onset of the differentiation protocol (Day 0) and repeatedly every 2 days until day 8 (D8) using 2 independent siRNAs (siAdig1 and siAdig2). (A, B) Representative images (10X) of AdipoRed staining of D8 adipocytes and quantification of AdipoRed intensity relative to siScramble (set at 100) (n=5). (C) mRNA expression of *Adig*, *Pparγ2*, *Glut4*, *Perilipin1* (*Plin1*), Leptin (*Lep*) and Adiponectin (*Adipoq*) relative to Scramble siRNA (set at 100) in cells treated with siAdig1 or siAdig2 (n=5-7). (D) Leptin and adiponectin levels secreted into culture media during a 48 hour (from D6 to D8) incubation. Data is expressed relative to the concentrations secreted in cells exposed to the scrambled siRNA (set as 100) (n=4-7).

(E-H) Knockdown of Adig was performed in 3T3-L1 adipocytes (from day 5 onwards) and repeated every 2 days until D8 of adipocyte differentiation when media was collected and cells were harvested for analysis, as described for A-D (n=3-5).

(I-J) Representative images (10X) of AdipoRed staining and quantification of its intensity in differentiated adipocytes (day 12 of differentiation) derived from stromal vascular cells isolated from inguinal adipose tissue of 10 week-old WT and *Adig*$^{-/-}$ male mice with (n= 3) or without Rosiglitazone (n= 2) treatment relative to WT (set at 100) (n=3 independent SVF isolations - 2 different replicates per experiment). (K) mRNA expression of *Adig*, *Pparγ2*, *Plin1*, *Lep* and *Adipoq* adipogenic markers relative to WT SVF or *Adig*$^{-/-}$ SVF, respectively (set at 100) (n= 2-3 independent SVF isolations - 2 different replicates per experiment).

Data is presented as mean ± SEM and analysed by 1-way ANOVA with Bonferroni multiple comparison post hoc testing (B-D and F-H p values obtained by compared to Scramble, and J, K compared to WT SVF). *p <0.05, **p < 0.01, ***p < 0.001, ****p < 0.0001. Relative Fluorescent Units (RFU). Differentiation (Diff), Rosiglitazone (Rosi).

In 3T3-L1 cells, *Adig* expression was effectively knocked down (KD) by repeated treatment (alternate days) with *Adig* siRNA (S3C, S3G). When the KD was initiated prior to the addition of differentiation cocktail, adipocyte differentiation was globally impaired as reflected by reduced lipid accumulation and reduced expression of several well-established markers of adipocyte differentiation (Figure S3A-C). This made it difficult to ascertain an independent effect of *Adig* KD on leptin expression or secretion so we also compared leptin expression in cells only exposed to *Adig* siRNA 5 days after the induction of differentiation. In this setting lipid accumulation and mRNA expression of typical adipocyte markers (*Pparγ2, Glut4* and *Plin1*) was unaffected whereas leptin mRNA was again significantly lower than in the control cells (Figure S3E-G). Furthermore, the levels of leptin detected in the media were lower in the KD cells (Figure S3D, S3H). Notably, secreted adiponectin levels, as well as mRNA expression, were also reduced in the *Adig* siRNA (Figure S3C, S3D and S3G, S3H), arguing against a leptin-specific effect in this experimental paradigm.

In order to confirm the 3T3-L1 results, we also isolated primary preadipocytes from the stromovascular fraction (SVF) of IngWAT derived from WT and *Adig*$^{-/-}$ mice. In these cells, lipid accumulation and gene expression analysis suggested that *Adig* was required for normal differentiation and that leptin expression was reduced in *Adig* null cells in keeping with this defect (Figure S3I-K). This defect could be effectively overcome by incubating the cells with rosiglitazone, a PPARγ agonist (Figures S3I-K). The mRNA expression of *Pparγ2, Plin1,* leptin (*Lep*) and adiponectin (*Adipoq*) showed a clear reduction in the *Adig* null derived SVF differentiated adipocytes in line with the lipid accumulation (Figure S3K).

These data collectively suggest that adipogenin deficiency impairs adipogenesis in cultured adipocytes and in adipocyte precursors derived from the SVF. Leptin expression is then also reduced in these cells but this may simply reflect the impairment in adipocyte differentiation rather than an additional direct effect.

Figure S4. ***Adig*** **gene, protein, phylogeny and membrane topology (Related to STAR methods section: Gene, protein, phylogeny and membrane topology (Bioinformatics))**

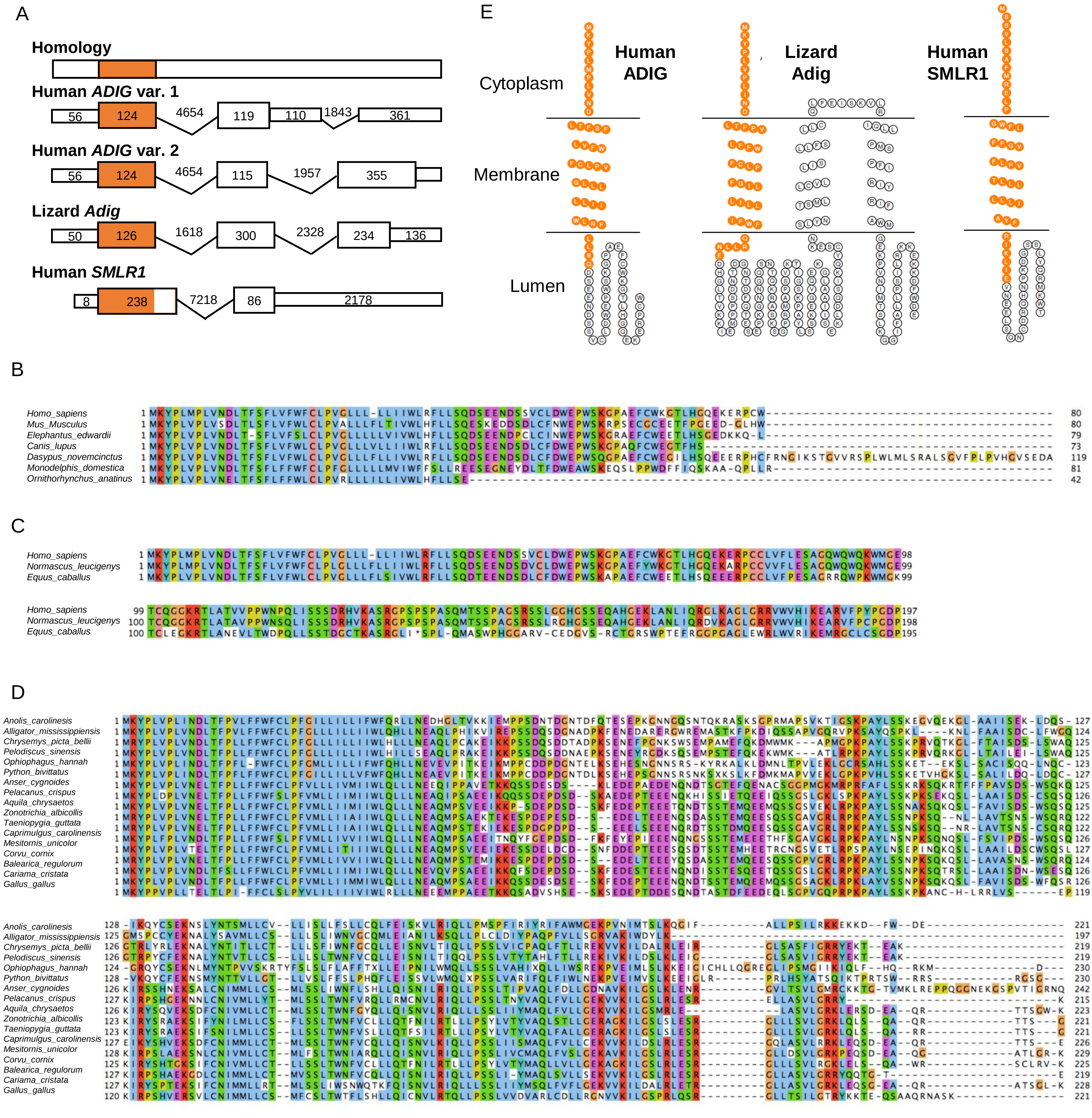

Figure S4. *Adig* gene, protein, phylogeny and membrane topology (Related to STAR methods section: Gene, protein, phylogeny and membrane topology (Bioinformatics))

(A) Structure of ADIG and SMLR1 genes from Mammalia and Sauria represented by genes from *Homo sapiens* and *Anolis carolinensis*. Boxes denote exons (coding and non-coding regions indicated as large and narrower) and lines for introns; the numbers stand for size in bp, the bar above indicates homology (orange). (B) Adig variant 1 Mammalian sequences alignment. (C) Alignment of mammalian examples of variants 2 that could be translated into proteins. (D) Adig alignment of Sauria. (E) Adig predicted structure and membrane topology in human and lizard, along with human SMLR1. Homologous sequences are highlighted in orange. The amino acids are colour coded according to their physical properties following the Jalview scheme (www.jalview.org).

Why adipogenin deficiency is associated with the phenotypes reported herein remains unclear, so to begin to address this question we performed a bioinformatics analysis of its evolution and amino acid sequence. Homology searches in genomic databases identified *ADIG* paralogues in Mammalia and Sauria but not in other vertebrates. *ADIG* is transcribed into two splice variants (Figure S4A). Variant 1 is the only one investigated experimentally thus far. It is translated into a short protein, the existence of which has been confirmed by immunoblotting (Ren et al., 2016b). Mammalian sequences of variant 1 are aligned in Figure S4B. Variant 2 gives a much larger transcript (confirmed by several cDNAs in transcriptomic databases) but the putative protein (197 amino acids) has not been investigated. Only the larger variant is transcribed from saurian genes. The gene structure of representative saurian *ADIG* and of the two mammalian variants is compared in Figure S4A. The shorter human variant is obtained through intron retention and almost immediate termination. Although the gene structures of the mammalian and saurian larger variants are similar, the homology exists only in the first exon. The rest of the larger mammalian variant diverged freely suggesting no or disappearing function. Examples of mammalian variants that could be translated into proteins are compared in Figure S4C. Others contain deletions leading to a premature termination. In sharp contrast, the homologous part in exon 1 is exceptionally strongly evolutionarily constrained indicating an important biological function. The saurian protein is constrained over the whole sequence with the N-terminus (first exon) showing the strongest conservation (Figure S4D).

The predicted structure and membrane topology of human and saurian ADIG is shown in Figure S4E. A signal peptide is not apparent and both mammalian variants have only one predicted transmembrane (TM) helix nearly identical to the saurian first TM helix (Figure S4E). Variant 1 only contains a short intra- and extracellular segment. The protein could be localized in the endoplasmic reticulum or lysosome according to the Localisation Signal Database (Negi et al., 2015).

Homology searches found one remote protein homologue Smlr1 (Small leucine-rich protein) of as yet unknown function predominantly expressed in adipocytes (proteinatlas.org). The membrane topology of the mammalian Adig is very similar to channel regulatory proteins phospholamban and sarcolipin (Shaikh et al., 2016). Saurian Adig shows a distant similarity to several transporters or channels. These initial observations tentatively hint at the potential function/s of Adig, without offering any clear insights into the observed physiological phenotypes.

Table S1. Oligonucleotide information (related to STAR Methods)

Oligo	Source	Identifier
Mouse Adig$^{+/+}$ genotyping Forward (WT): CTGACAGGTCTTCAGGAGCAG<br>Mouse Adig$^{+/+}$ Genotyping Reverse (WT): TACTGTGCATCCTCCCTTCTG	In this study	NA
Mouse Adig$^{-/-}$ genotyping Forward (KO): ACTTGCTTTAAAAAACCTCCCACA<br>Mouse Adig$^{-/-}$ Genotyping Reverse (KO): GGGTGTTTATGGAGACAGAATGCC	In this study	NA
Mouse Ob$^{+/+}$ genotyping Forward (WT): TGACCTGGAGAATCTCC<br>Mouse Ob$^{-/-}$ genotyping Forward (KO): TGACCTGGAGAATCTCT<br>Mouse Ob genotyping Reverse (WT and KO): CATCCAGGCTCTCTGGC	In this study	NA
Mouse Adipogenin Forward: TGTGGCTGTGAGGAGACATT<br>Mouse Adipogenin Reverse: CCACTTAGGCCCAGATGGTA	In this study	NA
Mouse Leptin Forward: CCAGGATGACACCAAAACCCT<br>Mouse Leptin Reverse: GATACCGACTGCGTGTGTGA	In this study	NA
Mouse Adiponectin Forward: GTTGCAAGCTCTCCTGTTCC<br>Mouse Adiponectin Reverse: ATCCAACCTGCACAAGTTCC<br>Mouse Adiponectin Probe: FAM-TCATGCCGAAGATGACGTTA-TAMRA	In this study	NA
Mouse Pparγ2 Forward: GATGCACTGCCTATGAGCACTT<br>Mouse PPARγ2 Reverse: AGAGGTCCACAGAGCTGATTCC<br>Mouse PPARγ2 Probe: FAM-AGAGATGCCATTCTGGCCCAC-TAMRA	In this study	NA
Mouse Glut4 Forward: TTATTGCAGCGCCTGAGTCT<br>Mouse Glut4 Reverse: GGGTTCCCCATCGTCAGAG<br>Mouse Glut4 Probe: FAM-TAAAACAAGATGCCGTCGGGTAMRA	In this study	NA
Mouse Ucp1 Forward: AGGCTTCCAGTACCATTAGGT<br>Mouse Ucp1 Reverse: CTGAGTGAGGCAAAGCTGATTT	In this study	NA
Mouse B2M Forward: ACTGATACATACGCCTGCAGAGTT<br>Mouse B2M Reverse: TCACATGTCTCGATCCCAGTAGA	In this study	NA
Mouse 36b4 Forward: AGATGCAGCAGATCCGCAT<br>Mouse 36b4 Reverse: GTTCTTGCCCATCAGCACC	In this study	NA
Mouse Hprt Forward: AGCCTAAGATGAGCGCAAGT<br>Mouse HPRT Reverse: GGCCACAGGACTAGAACACC	In this study	NA
Mouse CycA Forward: TTCCTCCTTTCACAGAATTATTCCA<br>Mouse CycA Reverse: CCGCCAGTGCCATTATGG<br>Mouse CycA Probe: FAM-ATTCATGTGCCAGGGTGGTGACTTTACAC-TAMRA	In this study	NA
Mouse Cpt1b Forward: TCTTCCCACCAGTCACTCAC<br>Mouse Cpt1b Reverse: CGGTACTTGGATTCTGTGCG	In this study	NA
Mouse Cpt1a Forward: CCTGGGCATGATTGCAAAG<br>Mouse Cpt1a Reverse: GCCACTCACGATGTTCTTCGT	In this study	NA
Mouse Pgc1a Forward: CACAACGCGGACAGAATTGAG<br>Mouse Pgc1a Reverse: TCACAGGTGTAACGGTAGGTGATG	In this study	NA
Mouse Pgc1b Forward: GGCCTTGTGTCAAGGTGGAT<br>Mouse Pgc1b Reverse: GGTGCTTATGCAGTTCCGTACA<br>Mouse Pgc1b Probe: AGACCCCCACACTGCCCCCTC	In this study	NA

Table S1. Oligonucleotide information (related to STAR Methods)

Oligo	Source	Identifier
Mouse Cd36 Taqman assay	Thermo Fisher Scientific	Cat#Mm01135198_m1
Mouse Pparα Taqman assay	Thermo Fisher Scientific	Cat#Mm00440939_m1
Mouse Acox1 Taqman assay	Thermo Fisher Scientific	Cat#Mm00443579_m1
Mouse Acot2 Taqman assay	Thermo Fisher Scientific	Cat#Mm01622461_s1
Mouse Pdk4 Taqman assay	Thermo Fisher Scientific	Cat#Mm01166879_m1
Mouse Fgf21 Taqman assay	Thermo Fisher Scientific	Cat# Mm00840165_g1
Mouse Acadl Taqman assay	Thermo Fisher Scientific	Cat#Mm00599660_m1
Mouse Acadm Taqman assay	Thermo Fisher Scientific	Cat#Mm 01323360_g1
ON-TARGETplus Non-targeting Pool (Scramble)	Dharmacon	Cat# D-001810-1
Silencer®Select Negative Control siRNA (Scramble)	Life Technologies	Cat# 4390847
ON-TARGETplus Adig	Dharmacon	Cat# J-041009-10-0002
Adig Silencer®Select (Mouse)	Life Technologies	Cat# s110859
